# Supplementary material for: Deep Learning Based Prediction of Gas Chromatographic Retention Indices for a Wide Variety of Polar and Mid-Polar Liquid Stationary Phases
Source: Int J Mol Sci. 2021 Aug 25;22(17):9194. doi: 10.3390/ijms22179194 (PMC8430916; doi:10.3390/ijms22179194)
Supplement: Supplementary file 1 [file ijms-22-09194-s001.zip › ijms-1342782-supplementary.pdf]

Supplementary Material: Deep learning based prediction of gas chromatographic retention indices for a wide variety of polar and mid-polar liquid stationary phases

Dmitriy D. Matyushin, Anastasia Yu. Sholokhova*, Aleksey K. Buryak

A.N. Frumkin Institute of Physical Chemistry and Electrochemistry, Russian Academy of Sciences

Pre-trained model parameters, source code, and corresponding instructions are available online:

https://doi.org/10.6084/m9.figshare.14602317

S1. Types of polar stationary phases

The names of stationary phases are given in accordance with the NIST 17 database. 20 the most popular in NIST 17 stationary phases are considered separately, and the rest of the stationary phases are grouped together in the "Other_polar" group. Many of the stationary phases that are listed separately are very similar, however, we follow the names and designations from NIST 17.

N	Name	N	Name	N	Name	N	Name
15	DB-Wax	20	PEG-20M	25	Innowax	30	Carbowax
16	Carbowax_20M	21	BP-20	26	Innowax_FSC	31	ZB-Wax
17	Supelcowax-10	22	FFAP	27	RTX-Wax	32	HP-Wax
18	OV-351	23	CP-Wax_52CB	28	PEG_4000	33	AT-Wax
19	HP-Innowax	24	HP-Innowax_FSC	29	DB-FFAP	34	Stabilwax
35	Other_polar						

S2. Overlaps between data sets

Data sets designations are used according to Table 1 from the main text of the article. Stereoisomers (*cis*/*trans* and optical) are considered as identical compounds, as well as isotopomers.

Data set	N_c	N_d	Overlap with NIST 17 (non-polar)*	Overlap with NIST 17 (polar)**
BPX50_2D (external test set)	329	332	305	56
BPX50_2D (test set)	167	168	152	73
BPX50_2D (training set)	359	359	330	145
DB-1701	36	36	35	33
DB-210	130	130	127	124
DB-624 (test set)	146	149	145	128
DB-624 (training set)	376	396	364	338
ESSOILS	383	427	383	381
FLAVORS	1098	1169	1054	1014
OV-17	171	192	162	153
SEDB624	130	130	127	124

N_c – the number of different compounds (stereoisomers, *cis*/*trans* isomers, and isotopomers are counted as identical compounds). N_d – the number of data records. * – the number of compounds from the data set for which the retention index for the non-polar stationary phase is given in the NIST 17 database. ** – the number of compounds for which the retention index for the polar stationary phase is given in the NIST 17 database.

Data set	BPX50_2D (external test set)	BPX50_2D (test set)	BPX50_2D (training set)	DB-1701	DB-210	DB-624 (test set)	DB-624 (training set)	ESSOILS	FLAVORS	OV-17	SEDB624
BPX50_2D (external test set)	329	3	5	2	5	3	8	5	8	1	5
BPX50_2D (test set)	3	167	11	2	8	4	15	12	19	6	8
BPX50_2D (training set)	5	11	359	3	17	15	29	25	34	19	17
DB-1701	2	2	3	36	3	4	14	14	27	8	3
DB-210	5	8	17	3	130	21	49	35	77	21	130
DB-624 (test set)	3	4	15	4	21	146	20	34	63	24	21
DB-624 (training set)	8	15	29	14	49	20	376	88	154	62	49
ESSOILS	5	12	25	14	35	34	88	383	183	91	35
FLAVORS	8	19	34	27	77	63	154	183	1098	79	77
OV-17	1	6	19	8	21	24	62	91	79	171	21
SEDB624	5	8	17	3	130	21	49	35	77	21	130

S3. Distributions of retention indices in data sets

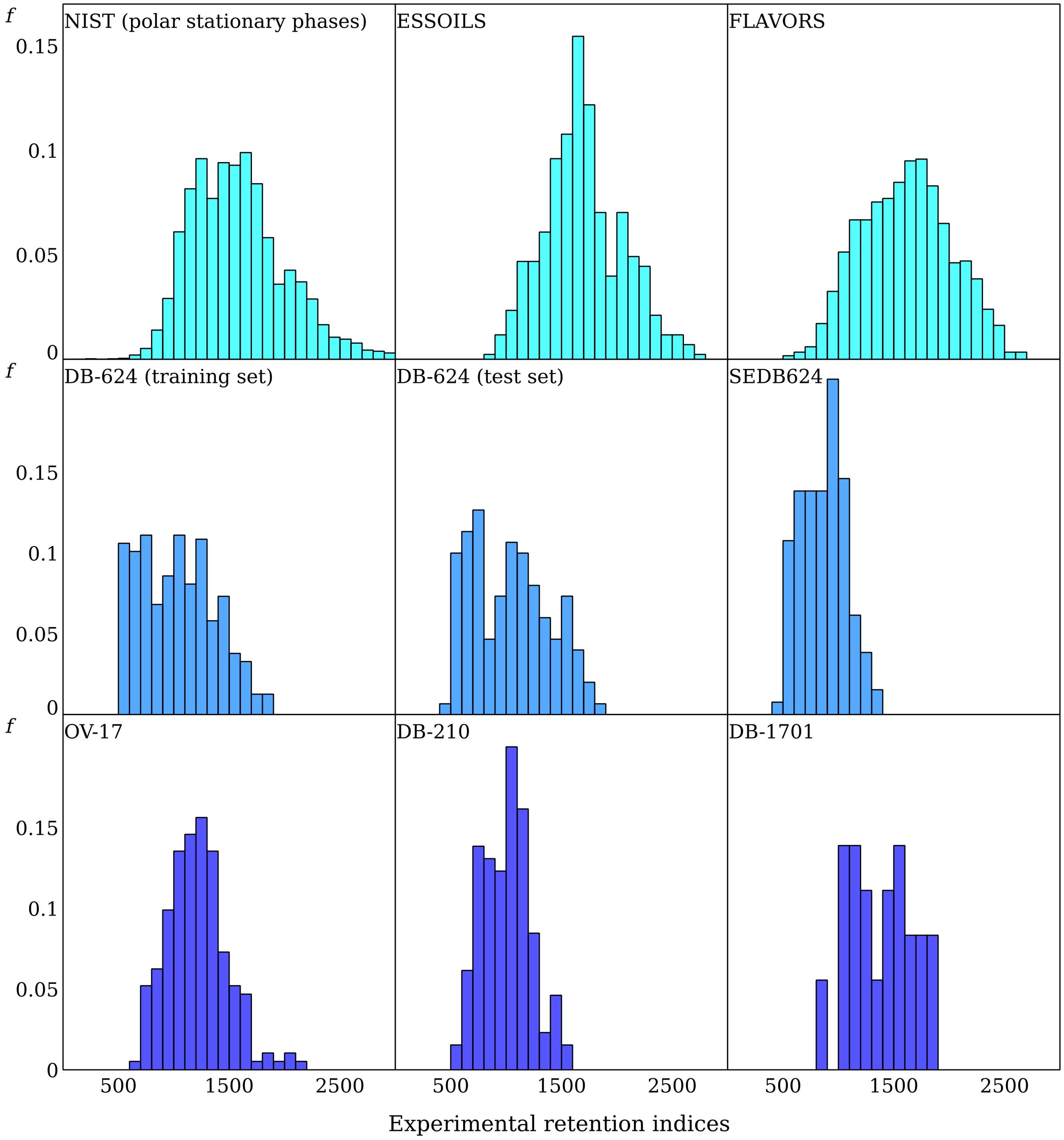

Distributions of retention indices (in terms of data entries) for the data sets considered in this work. *f* – the fraction of compounds in a bin. The first row – polar stationary phases, the second row – the DB-624 stationary phase, the third row – other mid-polar stationary phases.

S4. The correlation between log*k* and retention index

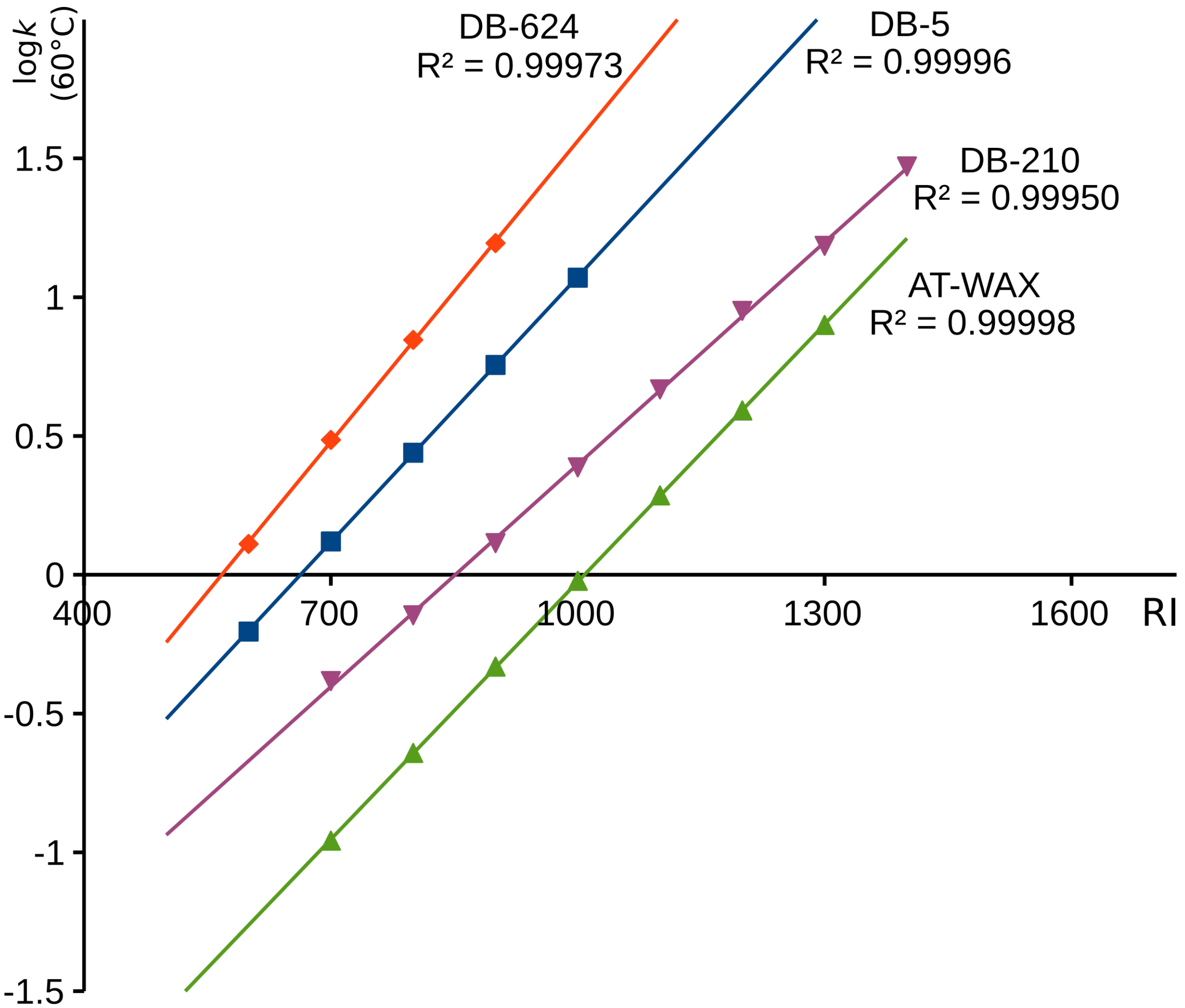

The correlation plot between log*k* (isothermal mode, 60°C) and the retention index for four stationary phases and some n-alkanes. The dependencies are nearly linear, coefficients of determination R^2 are given. Other compounds will also satisfy these linear dependencies due to definition of linear retention indices. Data (values of log*k* for four columns) were given from the following work:

Poole, C.F. Gas Chromatography System Constant Database for 52 Wall-Coated, Open-Tubular Columns Covering the Temperature Range 60–140 °C. *Journal of Chromatography A* **2019**, *1604*, 460482, doi:10.1016/j.chroma.2019.460482.
